# Monitoring of post-transplant *MLL*-PTD as minimal residual disease can predict relapse after allogeneic HSCT in patients with acute myeloid leukemia and myelodysplastic syndrome

**DOI:** 10.1186/s12885-021-09051-5

**Published:** 2022-01-03

**Authors:** Jun Kong, Meng-Ge Gao, Ya-Zhen Qin, Yu Wang, Chen-Hua Yan, Yu-Qian Sun, Ying-Jun Chang, Lan-Ping Xu, Xiao-Hui Zhang, Kai-Yan Liu, Xiao-Jun Huang, Xiao-Su Zhao

**Affiliations:** 1 Beijing Key Laboratory of Hematopoietic Stem Cell Transplantation, Peking University People’s Hospital, Peking University Institute of Hematology, National Clinical Research Center for Hematologic Disease, No 11 Xizhimen South Street, Beijing, 100044 China; 2 Research Unit of Key Technique for Diagnosis and Treatments of Hematologic Malignancies, Chinese Academy of Medical Sciences, 2019RU029, Beijing, China; 3 Collaborative Innovation Center of Hematology, Peking University, Beijing, China; 4 Peking-Tsinghua Center for Life Sciences, Beijing, 100044 China

**Keywords:** *MLL*-PTD, Minimal residual disease, Allogeneic hematopoietic stem cell transplantation, Relapse

## Abstract

**Background:**

The mixed-lineage leukemia (*MLL*) gene is located on chromosome 11q23. The MLL gene can be rearranged to generate partial tandem duplications (MLL-PTD), which occurs in about 5-10% of acute myeloid leukemia (AML) with a normal karyotype and in 5-6% of myelodysplastic syndrome (MDS) patients. Allogeneic hematopoietic stem cell transplantation (allo-HSCT) is currently one of the curative therapies available for AML and MDS with excess blasts (MDS-EB). However, how the prognosis of patients with high levels of *MLL*-PTD after allo-HSCT, and whether *MLL*-PTD could be used as a reliable indicator for minimal residual disease (MRD) monitoring in transplant patients remains unknown. Our study purposed to analyze the dynamic changes of *MLL*-PTD peri-transplantation and the best threshold for predicting relapse after transplantation.

**Methods:**

We retrospectively collected the clinical data of 48 patients with *MLL*-PTD AML or MDS-EB who underwent allo-HSCT in Peking University People’s Hospital. The *MLL*-PTD was examined by real-time quantitative polymerase chain reaction (RQ-PCR) at the diagnosis, before transplantation and the fixed time points after transplantation. Detectable *MLL*-PTD/ABL > 0.08% was defined as *MLL*-PTD positive in this study.

**Results:**

The 48 patients included 33 AML patients and 15 MDS-EB patients. The median follow-up time was 26(0.7-56) months after HSCT. In AML patients, 7 patients (21.2%) died of treatment-related mortality (TRM), 6 patients (18.2%) underwent hematological relapse and died ultimately. Of the 15 patients with MDS-EB, 2 patients (13.3%) died of infection. The 3-year cumulative incidence of relapse (CIR), overall survival (OS), disease-free survival (DFS) and TRM were 13.7 ± 5.2, 67.8 ± 6.9, 68.1 ± 6.8 and 20.3% ± 6.1%, respectively. ROC curve showed that post-transplant *MLL*-PTD ≥ 1.0% was the optimal cut-off value for predicting hematological relapse after allo-HSCT. There was statistical difference between post-transplant *MLL*-PTD ≥ 1.0% and *MLL*-PTD < 1.0% groups (3-year CIR: 75% ± 15.3% vs. 0%, *P* < 0.001; 3-year OS: 25.0 ± 15.3% vs. 80.7% ± 6.6%, *P* < 0.001; 3-year DFS: 25.0 ± 15.3% vs. 80.7 ± 6.6%, *P* < 0.001; 3-year TRM: 0 vs. 19.3 ± 6.6%, *P* = 0.277). However, whether *MLL*-PTD ≥ 1% or *MLL*-PTD < 1% before transplantation has no significant difference on the prognosis.

**Conclusions:**

Our study indicated that *MLL*-PTD had a certain stability and could effectively reflect the change of tumor burden. The expression level of *MLL*-PTD after transplantation can serve as an effective indicator for predicting relapse.

## Background

Acute myeloid leukemia (AML) is a highly malignant hematopoietic system disease and myelodysplastic syndrome (MDS) is a type of heterogeneous myeloid malignancies and frequently progress to AML [1–4]. In previous studies, molecular genetic aberrations have become important approaches for minimal residual disease (MRD) detection for AML and MDS. Especially, the polymerase chain reaction (PCR)-based gene detection has been proven to be an effective MRD monitoring method for AML patients [5–7]. However, more than half of AML cases still lack effective specific MRD molecular markers [5].

The mixed-lineage leukemia (*MLL*) gene, also named lysine (K)-specific methyltransferase 2A (KMT2A), is located on chromosome 11q23. Genetic alterations of the MLL gene are usually associated with the development of acute leukemia [8]. The MLL gene may be rearranged to generate partial tandem duplications (*MLL*-PTD), which usually spans exons 2 to 6, 2 to 7, and 2 to 8, or exons 3-9, exons 3-10, exons 3-11, or exons 3-10 and exons 3-11 at the molecular level [8–11]. *MLL-*PTD has been detected in approximately 5-10% of AML and 5-6% of MDS patients [12–14]. Low level of *MLL-*PTD (< 0.08%) may also be present in the blood and bone marrow of healthy individuals [5]. Previous reports support that polymerase chain reaction (PCR)-based *MLL-*PTD is a reliable MRD marker and is associated with poor prognosis [5, 12–15]. For chemotherapy patients, a higher *MLL-*PTD level at initial diagnosis predicts a lower incidence of chemotherapy complete remission (CR) and a lower survival rate [13]. The dynamic changes of chemotherapy patients also show that *MLL-*PTD levels within the first 6 months after the start of therapy are useful for early risk assessment of AML patients, and that a reduction of *MLL-*PTD level ≥ 2 log is a good prognostic factor for overall survival [5]. Furthermore, compared with healthy donors, *MLL-*PTD level have no difference from that of non-transplanted patients in continuous CR, while was significantly higher than that of transplanted patients in continuous CR [15]. Taken together, these findings support that *MLL-*PTD is a specific clinical prognostic marker in the initial diagnosis and chemotherapy for AML patients. However, there are few reports on the dynamics of *MLL-*PTD peri-transplantation, especially after transplantation. Thus, whether *MLL-*PTD could be used as a stable and reliable MRD marker in the process of transplantation and whether there is an optimal value of *MLL-*PTD to predict relapse after transplantation will be explored for the first time in our study.

In this study, we investigated a consecutive cohort of 33 AML and 15 MDS patients with *MLL-*PTD who received allo-HSCT at our institute. Most *MLL-*PTD MDS cases are classified as MDS with excess blasts (MDS-EB) [16]. Our study purposed to analyze the dynamic changes of *MLL-*PTD peri-transplantation and the best threshold for predicting relapse after transplantation.

## Methods

### Patients

The consecutive patients diagnosed with *MLL-*PTD expression> 0.08% AML or MDS undergoing allo-HSCT between January 2015 and March 2019 at the Peking University People’s Hospital, Institute of Hematology were enrolled in this study. The patients’ data were updated until September 30, 2020. The institutional review board at the hospital approved the protocol, and all patients or their guardians signed consent forms approved by the institutional review board.

### Transplantation protocol

All the patients in this study received myeloablative conditioning regimens. Haploidentical HSCT (haplo-HSCT) and matched sibling donor transplantation (MSDT) were performed according to protocols reported previously by our institute [17, 18]. The conditioning regimen for MSDT patients is: Cytarabine (Ara-C) 2 g/m^2^/d i.v. for 1 day, cyclophosphamide (CTX) 1.8 g/m^2^/d for 2 days, busulfan (BU) 0.8 mg/kg i.v., q.i.d. for 3 days, and nitrosourea (Simustine, MeCCNU) 250 mg/kg for 1 day. The conditioning regimen for haplo-HSCT patients is: Ara-C 4 g/m^2^/d i.v. for 2 days, CTX 1.8 g/m^2^/d for 2 days, BU 0.8 mg/kg i.v., q.i.d. for 3 days, and MeCCNU 250 mg/kg for 1 day, and thymoglobulin (ATG, Sang Stat, Lyon, France) 2.5 mg/kg/d i.v. for 4 days.

### Donor Lymphocyte Infusion (DLI)

Prophylactic DLI was administered for patients in relapse or no remission (NR) state before transplantation. The indications for DLI included hematological leukemia relapse, receiving chemotherapy followed by DLI, or positive MRD detection as previously described [19].

### Detection of MRD

In this study, MRD was evaluated by Flow Cytometry (FCM) [20], the expression level of *WT1* and *MLL-*PTD determined by RQ-PCR. The pre-transplant FCM, *MLL-*PTD and *WT1* were performed using bone marrow (BM) samples within a month before the transplant as a routine. The post-transplant scheduled time points were + 1, + 2, + 3, + 4.5, + 6, + 9, and + 12 months post-HSCT and every 6 months thereafter.

The patients were analyzed for the presence of MLL-PTD at the MLL gene locus, as described previously [13, 15]. Briefly, *MLL* primers and hybridization probes were placed in exons 8-10 and 3 of the *MLL* gene, allowing for detection of *MLL-*PTD with exon 8/exon 3 fusion, exon 9/exon 3 fusion, or exon 10/exon 3 fusion. The transcript level was calculated as target transcript copies/*ABL* copies in percentages. Detectable *MLL-*PTD/*ABL* > 0.08% was defined as *MLL-*PTD positive [13]. The *WT1* was detected as described previously and a *WT1* transcript level less than 0.60% was defined as negative [21].

### Definitions and assessments

The day of neutrophil engraftment was defined as the first day of 3 consecutive post-transplantation days on which the absolute neutrophil count (ANC) exceeded 500/μL. Patients who survived at least 28 days were considered to have had successful engraftment. The criteria for grading acute graft versus host disease (aGVHD) have been previously published [22, 23]. CR was defined as hematological CR that is, < 5% BM blasts, the absence of blasts in peripheral blood, the absence of extramedullary disease, an ANC > 1.0 × 10^9^/L, and a platelet count > 100 × 10^9^/L with no red cell transfusions. Hematological relapse was defined by morphologic evidence of disease in the peripheral blood, marrow, or extramedullary sites.

### Statistical analysis

The primary study end point was the cumulative incidence of relapse (CIR). The secondary end points were the OS, disease-free survival (DFS) and treatment-related mortality (TRM). CIR, OS, DFS and TRM were defined as previously described [24]. Summary statistics, such as proportions, medians and ranges, were used to describe the patient characteristics and outcomes. The associations between *MLL-*PTD expression and post-transplantation outcomes were analyzed by the Kaplan-Meier method. Differences in CIR, DFS, OS and TRM between groups were calculated using the log-rank test. A two-sided *P* value of 0.05 was considered statistically significant. The independence of categorical parameters was calculated using the chi-square test or Fisher exact test, and the distribution of continuous variables was calculated using the Mann-Whitney U-test. All statistical analyses were performed using SPSS 23.0 (Chicago, IL, USA).

## Results

### Patients characteristics

**Fig. 1:**
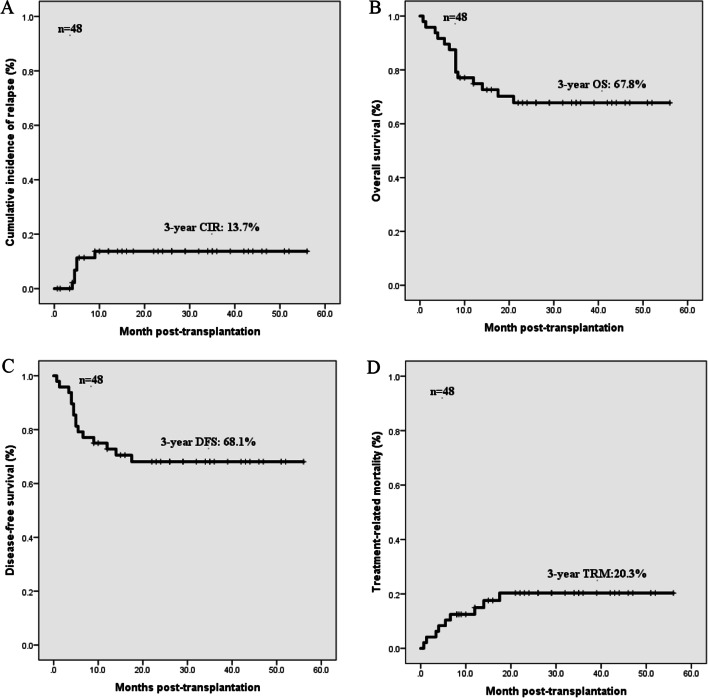
Cumulative incidence of relapse, overall survival, disease-free survival and treatment-related mortality of 48 *MLL*-PTD patients after allo-HSCT

A total of 33 AML patients included 13 males and 20 females, with a median age of 42 years (10-57 years) and 15 MDS-EB patients included 11 males and 4 females, with a median age of 51 years (4-60 years). The median follow-up time was 26 (0.7-56) months after HSCT. Patient characteristics are shown in Table 1. Of these 33 AML patients, 31 patients had gotten CR after chemotherapy, and 2 patients had gotten NR after 3 courses of chemotherapy. And 5 MDS-EB patients receiving chemotherapy including decitabine had gotten CR pre-transplantation. All patients had neutrophil engraftment, and 39 patients had platelet engraftment. Of the 33 patients with AML, 7 patients (21.2%) died of TRM and 6 patients (18.2%) underwent hematological relapse who died ultimately. The median hematological relapse time was 4.8 months (range 4-9 months) after HSCT in 6 relapsed patients. Of the 15 patients with MDS-EB, 2 patients (13.3%) died of infection. In addition, all enrolled patients had a 3-year CIR of 13.7% ± 5.2%, 3-year OS of 67.8% ± 6.9%, 3-year DFS of 68.1% ± 6.8% and 3-year TRM of 20.3% ± 6.1% (Fig. 1).

**Table 1:** Characteristics of acute myeloid leukemia and myelodysplastic syndrome patients

Characteristic	AML ***N*** = 33	MDS-EB1/2 ***N*** = 15
Median age at allo-HCT, years (range)	42 (10–57)	51 (4–60)
Gender, n (%)		
Male	13 (39.4%)	11 (73.3%)
Female	20 (60.6%)	4 (26.7%)
Chromosome normal, n (%)	23 (69.7%)	9 (60.0%)
FLT3-ITD mutation, n (%)		
Yes	10 (30.3%)	0
No	23 (69.7%)	15 (100%)
NPM1 mutation, n (%)	0	0
Risk category		
Favorable	0	0
Intermediate	33	15
Adverse	0	0
Median WT1 expression level at initial diagnosis	25.25 (0.23-83.20)	18.80 (1.40-53.50)
No remission before transplant, n (%)	2 (6.1%)	1 (6.7%)
Donor type, n (%)		
HLA-matched sibling	7(21.2%)	5(33.3%)
Haploidentical	26(78.8%)	10(66.7%)
ABO blood type match, n (%)		
Compatible	17 (51.5%)	7 (46.7%)
Incompatible	16 (48.5%)	8 (53.3%)
Conditioning regimen, n (%)		
Chemotherapy based	33 (100%)	15 (100%)
TBI based	0	0
Cell compositions in allografts		
Median MNC, × 10^8^/kg (range)	7.82 (6.04-10.86)	8.54 (6.10-10.86)
Median CD34+ count, × 10^6^/kg (range)	2.32 (0.27-6.67)	1.89 (0.84-5.34)
Granulocyte engraftment time, day (range)	13 (8-25)	13 (11-19)
Platelet engraftment time, day (range)	14 (10-74)	13 (10-53)
II–IV°aGVHD	8 (24.2%)	1 (6.7%)
aGVHD	18 (54.5%)	4 (26.7%)
cGVHD	5 (15.2%)	5 (33.3%)
DLI after transplant, n (%)		
For relapse prevention	2 (6.1%)	0
For intervention	4 (12.1%)	2 (13.3%)
Prognosis, n (%)		
Relapse	6 (18.2%)	0
Treatment-related death	7 (21.2%)	2 (13.3%)
Relapse death	6 (18.2%)	0

*AML* acute myeloid leukemia, *MDS* myelodysplastic syndrome, *HLA* human leukocyte antigen, *TBI* total body irradiation, *MNC* mononuclear cell, *aGVHD* acute graft versus host disease, *cGVHD* chronic graft versus host disease, *DLI* donor lymphocyte infusion

### Dynamic changes of *MLL-*PTD before and after transplantation

Observing the changes in the expression level of *MLL-*PTD at different time points peri-transplantation is helpful to analyze the stability of *MLL-*PTD as an MRD indicator in the transplantation system. Our results showed that the *MLL-*PTD level before transplantation was significantly lower than that at the initial diagnosis, but there were still 37 cases were *MLL-*PTD positive before transplantation, and 33 of 37 cases became negative within post-transplant 1 month. However, during our follow-up period, 25 cases eventually occurred post-transplant *MLL-*PTD positive. The median *MLL-*PTD level in all enrolled patients was decreased by around 35 folds after transplantation compared with that of pre-transplant CR status and was similar to the healthy controls (Table 2). Furthermore, among the 6 relapsed patients after transplantation, 3 of them maintained *MLL-*PTD at the healthy level (< 0.08%) within a month after transplantation. But before relapse, the *MLL-*PTD level of these 3 patients gradually increased (> 0.08%) and reached the highest level at the time of relapse. The *MLL-*PTD level of the other 3 relapsed patients continuously remained > 0.08% after transplantation, and the *MLL-*PTD levels of these 3 patients suddenly increased by hundreds of times before relapse.

**Table 2:** Comparison of *MLL*-PTD and *WT1* at the initial diagnosed and peri-transplant patients

	MLL-PTD > 0.08% (n/total tests, positive rate)	Median level of MLL-PTD > 0.08% (range, %)	Median level of MLL-PTD (range, %)	WT1 > 0.6% (n/total tests)	Median level of WT1 > 0.6% (range, %)	*P* value (MLL-PTD+ vs. WT1+)
**The initial diagnosis**	48/48(100%)	30.30 (1.20-631.00)	30.30 (1.20-631.00)	44/47(93.6%)	26.20 (0.82-83.20)	0.233
**Pre-transplantation**	37/48(68.8%)	6.10 (0.10-414.10)	1.70 (0.017-414.10)	28/47(59.6%)	6.20 (0.88-53.50)	0.351
**Post-transplantation**						
+ 1 month	8/43(18.6%)	0.115 (0.083-0.73)	0.046 (0.01-0.73)	1/46(2.2%)	0.82	0.027
+ 2 month	12/44(27.3%)	0.21 (0.09-0.82)	0.047 (0-0.82)	3/45(6.7%)	0.86 (0.74-2.4)	0.009
+ 3 month^a^	13/45(28.9%)	0.28 (0.086-104.70)	0.05 (0-104.70)	6/46(13.0%)	1.50 (0.75-32.70)	0.063
+ 4.5 month^a^	8/38(21.1%)	1.30 (0.082-55.30)	0.0515 (0-55.30)	9/39(23.1%)	3.90 (0.81-44.10)	0.524
+ 6 month^a^	11/39(28.2%)	1.40 (0.096-101.30)	0.053 (0.015-101.30)	12/39(30.8%)	1.30 (0.60-80.90)	0.500
+ 9 month^a^	5/27(18.5%)	0.09 (0.08-0.11)	0.0445 (0-1.00)	3/34(8.8%)	0.71 (0.63-0.74)	0.231
+ 12 month	1/30(3.3%)	0.45	0.049 (0-0.45)	5/32(15.6%)	0.88 (0.72-1.00)	0.113

^a^Patients underwent hematological relapse at that time point

### The effect of MLL-PTD level before and after transplantation on prognosis

**Fig. 2:**
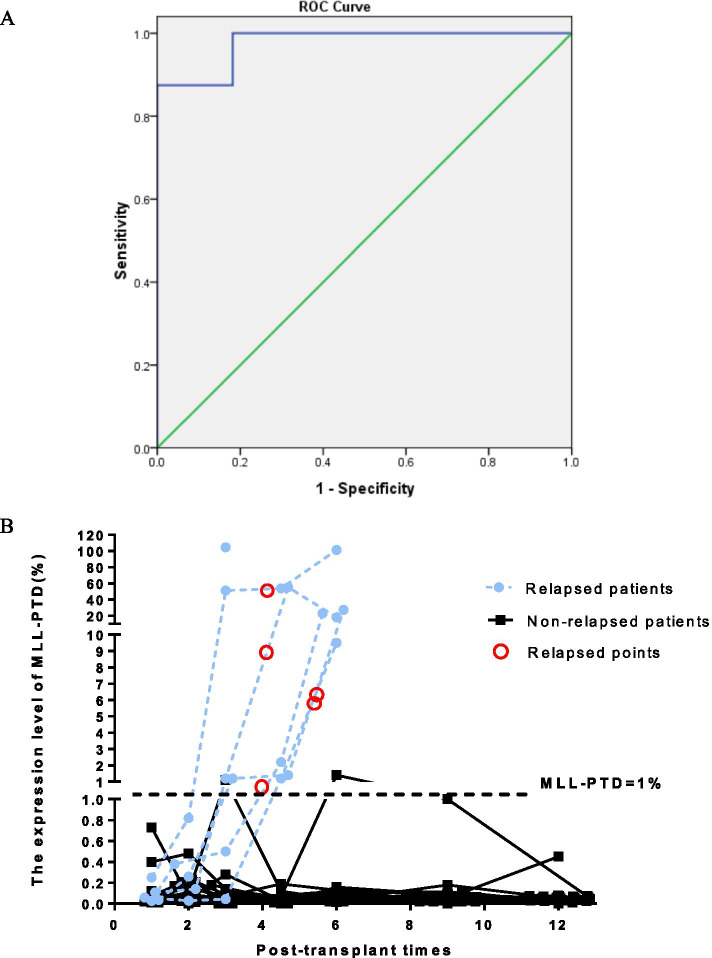
The curve of *MLL*-PTD expression levels post-transplantation. **A** Receiver operating characteristic (ROC) curve of *MLL*-PTD expression post-transplantation (AUC = 0.977, *P* < 0.001). **B** The level changes of post-transplant *MLL*-PTD

**Fig. 3:**
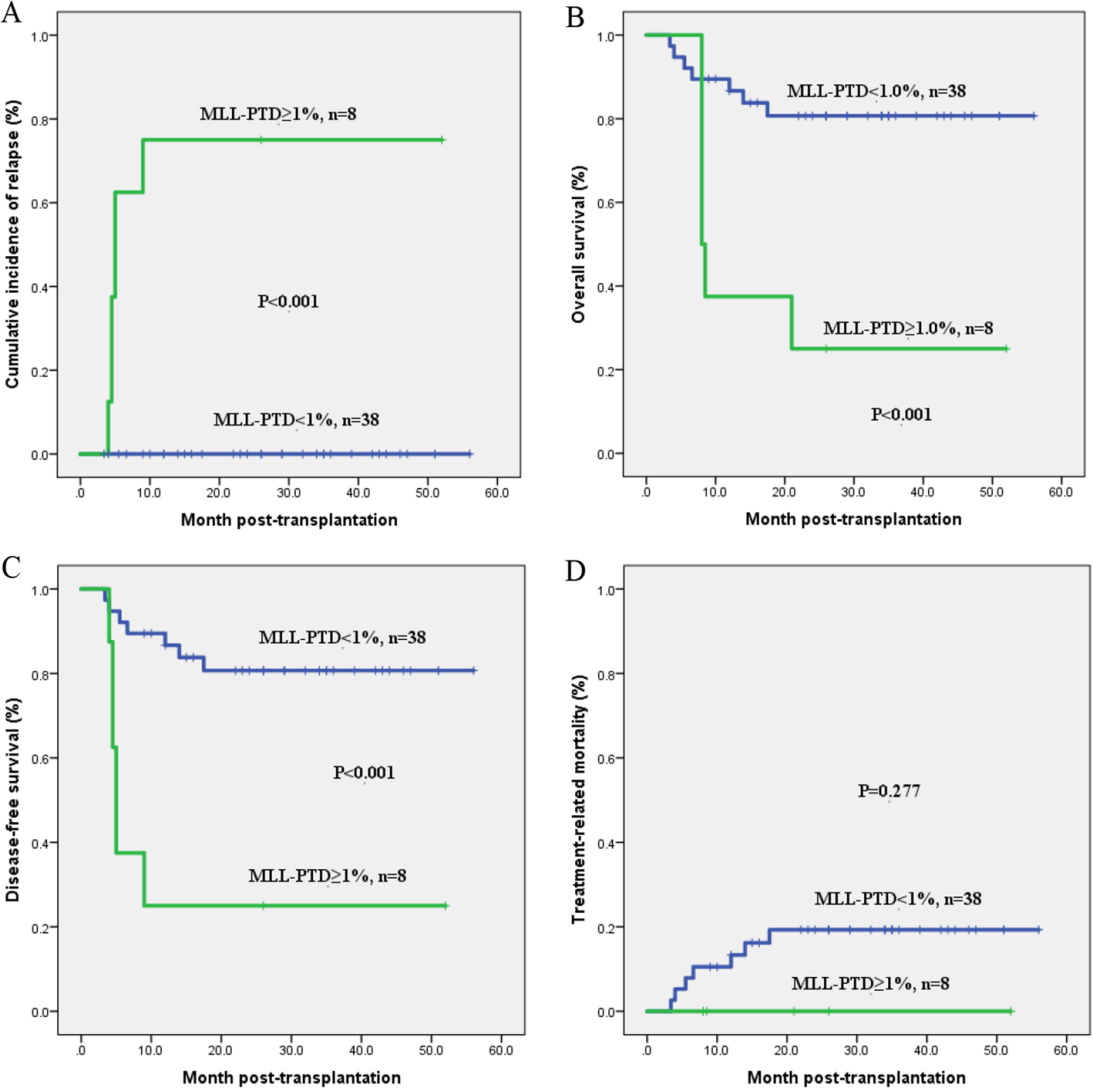
Kaplan-Meier survival curves analysis of patients between *MLL*-PTD < 1% and *MLL*-PTD ≥ 1% after transplantation

Having analyzed the dynamic changes above which peri-transplant *MLL-*PTD can stably reflect the disease state we next studied the optimal threshold of post-transplant *MLL-*PTD for relapse. Our previous study shows that patients with *MLL-*PTD/*ABL* ≥ 1% based on initial diagnosis have a poor clinical prognosis [13]. In order to explore whether *MLL-*PTD could be used as a MRD marker after transplantation, we performed a receiver operating characteristic (ROC) with the highest expression level of post-transplant *MLL-*PTD before hematological relapse in all patients to determine the optimal cut-off value to predict relapse. The area under the ROC curve value was 0.977 (*P* < 0.001, Fig. 2A). The optimal cut-off value was *MLL-*PTD/*ABL* = 1.0%. And as shown in Fig. 2B, most post-transplant patients with *MLL-*PTD maintained a low level of expression, only 8 patients had *MLL-*PTD ≥ 1%, and 6 of the 8 patients eventually relapsed, which also implied the importance of *MLL-*PTD ≥ 1% in predicting relapse after transplantation. Based on the optimal cut-off value, we divided the post-transplant patients into two groups of *MLL-*PTD/*ABL* < 1% and *MLL-*PTD/*ABL* ≥ 1% to analyzed the prognostic difference. Our study found that the group of *MLL-*PTD/*ABL* ≥ 1.0% had higher 3-year CIR (75 ± 15.3% vs. 0%, *P* < 0.001, Fig. 3A), and lower 3-year OS (25.0 ± 15.3% vs. 80.7% ± 6.6%, *P* < 0.001, Fig. 3B) and 3-year DFS (25.0 ± 15.3% vs. 80.7 ± 6.6%, *P* < 0.001, Fig. 3C) compared with that of group of *MLL-*PTD/*ABL* < 1%. However, there was no statistical difference between the two groups in TRM (*P* > 0.05, Fig. 3D).

**Fig. 4:**
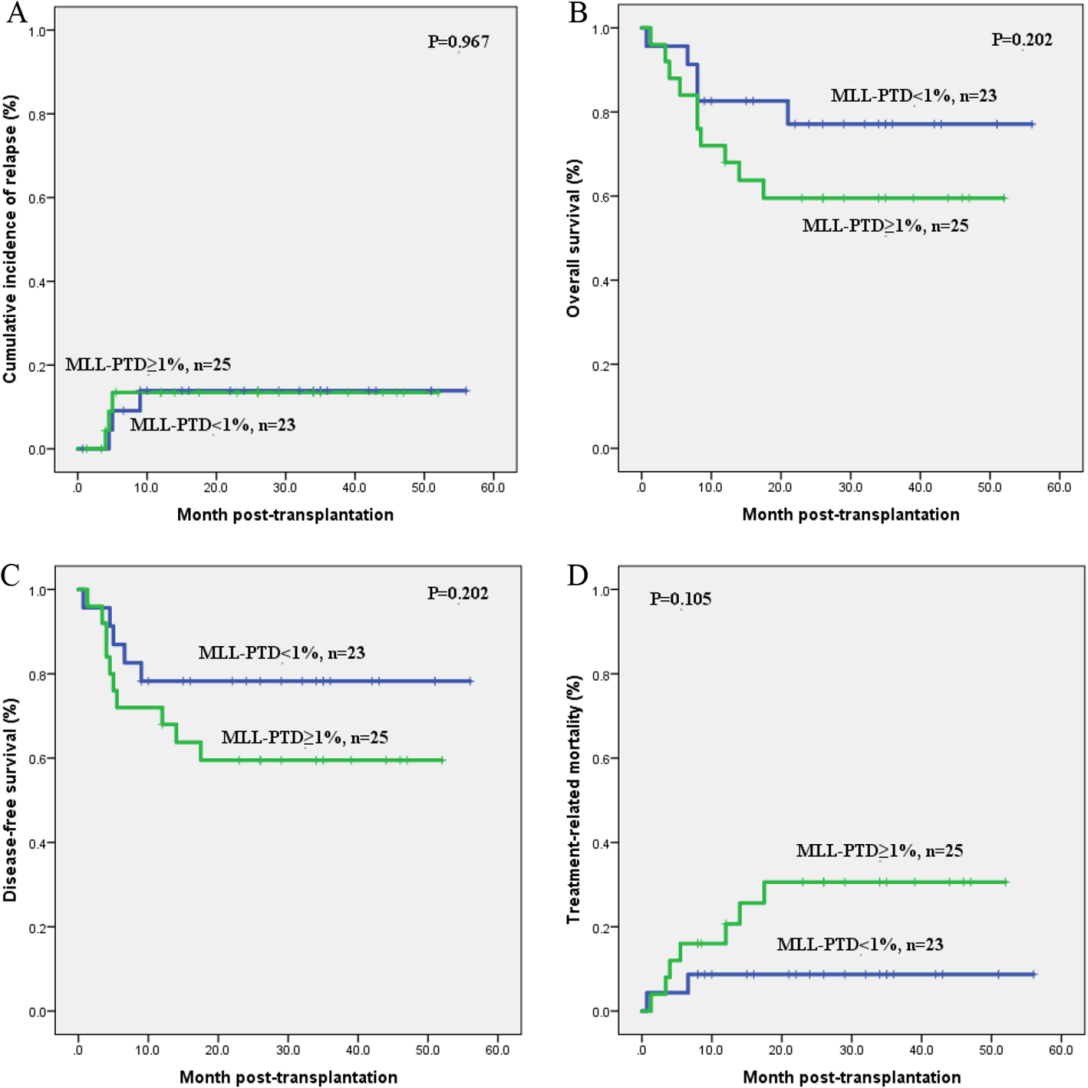
Kaplan-Meier survival curves analysis of patients between *MLL*-PTD < 1% and *MLL*-PTD ≥ 1% before transplantation

Both at the initial diagnosis and post-transplantation, it was analyzed that *MLL-*PTD = 1% was the optimal cut-off value, which implied that *MLL-*PTD/ABL = 1% was of important value in predicting prognosis. Therefore, we further analyzed whether *MLL-*PTD*/ABL* ≥ 1% before transplantation also indicated a poor prognosis after transplantation. However, our results showed that there was no statistical difference in prognosis between the *MLL-*PTD*/ABL* ≥ 1% and *MLL-*PTD*/ABL* < 1% group based on the level of *MLL-*PTD before transplantation (All *P* > 0.05, Fig. 4A-D), but the group of *MLL-*PTD*/ABL* ≥ 1% tended to have lower OS (*P* = 0.202, Fig. 4B), DFS (*P* = 0.202, Fig. 4C), and have a higher TRM(*P* = 0.105, Fig. 4D) compared with that of *MLL-*PTD*/ABL* < 1% group .

### Factors affecting the prognosis of transplant patients with *MLL-*PTD

Factors affecting the prognosis were analyzed, including transplantation age, gender, disease type, donor type, blood type compatibility (Table 3). There was no statistical difference in TRM (*P* = 0.675), CIR (*P* = 0.115), DFS (*P* = 0.151) and OS (*P* = 0.157) between AML and MDS-EB. Among the 12 patients who received MSDT, 2 (16.7%) patients underwent hematological relapse both at 5 months after HSCT, and 1 patient died of pneumonia at 5.5 months. Among 36 patients who received haplo-HSCT, 4 patients (11.1%) underwent hematological relapse at a median of 4.5 months (range, 4-9 months) after HSCT, and 8 patients (22.2%) died due to TRM at a median of 5.3 months (range, 0.7-17.5 months). Based on the results of the analysis, it seemed that patients who received haplo-HSCT could achieve comparable outcomes compared to those who underwent MSDT (TRM: *P* = 0.271; CIR: *P* = 0.653; DFS: *P* = 0.544; OS: *P* = 0.560). The factor analysis of *MLL-*PTD level before and after transplantation showed that there was no statistical difference in pre-transplant *MLL-*PTD level. And post-transplant group of *MLL-*PTD*/ABL* ≥ 1% had a higher CIR, a lower OS and a lower DFS than that of group of *MLL-*PTD*/ABL* < 1% (all *P* < 0.001). In addition, other factors such as age, pre-transplant FCM, *WT1* status and prophylactic DLI have no significant impact on prognosis. The ABO blood type and *FLT3-ITD* mutation at first diagnosis were important risk factors of CIR and OS after transplantation, respectively. Incompatible ABO blood type indicated a higher CIR than that of compatible ABO blood type, and patients with *FLT3-ITD* mutation had a low OS than that of without *FLT3-ITD* (Table 3).

**Table 3:** Univariate analysis of the variables affecting hematological TRM, CIR, DFS and OS in patients with *MLL*-PTD after allo-HSCT

Variables	Number (n,%)	***P*** value			
		TRM	CIR	DFS	OS
**Age of recipient**		0.965	0.291	0.410	0.442
<50 years	31(64.6%)				
≥ 50 years	17(35.4%)				
**Underlying disease**		0.675	0.115	0.151	0.157
AML	33(68.8%)				
MDS-EB1/2	15(31.2%)				
**ABO compatibility**		0.264	0.009	0.38	0.484
Compatible	24(50.0%)				
Incompatible	24(50.0%)				
**Donor type**		0.271	0.653	0.544	0.560
HLA-matched sibling	12(25.0%)				
Haploidentical	36(75.0%)				
**Prophylactic DLI**	2(4.2%)	0.325	0.591	0.735	0.702
**FLT3-ITD positive**	10(20.8%)	0.067	0.868	0.068	0.041
**Pre-transplantation FCM**		0.056	0.504	0.291	0.232
Negative	23(47.9%)				
Positive	25(52.1%)				
**Pre-transplantation WT1**		0.339	0.166	0.843	0.854
WT1 < 0.6%	19(40.4%)				
WT1 ≥ 0.6%	28(59.6%)				
**Pre-transplantation MLL-PTD**		0.105	0.967	0.202	0.202
MLL-PTD/ABL ≥ 1.0%	25(52.1%)				
MLL-PTD/ABL<1.0%	23(47.9%)				
**Post-transplantation MLL-PTD**		0.277	< 0.001	< 0.001	< 0.001
MLL-PTD/ABL ≥ 1.0%	8(16.7%)				
MLL-PTD/ABL<1.0%	38(79.2%)				

*TRM* treatment-associated mortality, *CIR* cumulative incidence of relapse, *DFS* disease-free survival, *OS* overall survival, *HLA* human leukocyte antigen, *allo-HSCT* allogeneic hematopoietic stem cell transplantation, *DLI* donor lymphocyte infusion, *MLL-PTD* mixed lineage leukemia-partial tandem duplication, *AML* acute myeloid leukemia, *MDS* myelodysplastic syndrome

### Comparison of *MLL-*PTD and other MRD parameters

After transplantation, 8 patients were detected *MLL-*PTD*/ABL* ≥ 1.0% at a median of 3 months. Of the 8 patients, 7 patients were simultaneously (5 patients) or subsequently (2 patients) MRD positive detected by FCM at a median of 4.25 months (range,3-12 months), and 6 patients ultimately progressed to hematological relapse at a median of 2 months (range, 0.25–6 months) from the first time *MLL-*PTD*/ABL* ≥ 1.0%, half of whom receiving chemotherapy plus DLI. Finally, 2 patients receiving chemotherapy plus DLI became MRD negative gradually.

*WT1* has been confirmed in previous studies to be an effective indicator of MRD monitoring and implementing interventions [21]. In order to analyze the specificity and sensitivity of *MLL-*PTD compared with *WT1*, we showed in Table 2 the dynamic changes of expression of *MLL-*PTD and *WT1* at the initial diagnosis and different time points before and after transplantation. All 6 relapsed patients were detected *MLL-*PTD positive prior to relapse, while only 4 patients were detected positive for *WT1*. As shown in Table 2, the expression levels of *MLL-*PTD and *WT1* both changed with the tumor burden. However, within post-transplant 3 months, *MLL-*PTD seemed be more sensitive than *WT1* for MRD monitoring (*P*_*+ 1 month*_ = 0.027; *P*_*+ 2 month*_ = 0.009; *P*_*+ 3 month*_ = 0.063).

## Discussion

*MLL-*PTD is a special *MLL* rearrangement gene. No report had focused on the predictive significance of peri-transplant *MLL-*PTD expression on leukemia relapse after transplantation. In our retrospective study, results showed dynamic changes of *MLL-*PTD peri-transplantation, and the post-transplant *MLL-*PTD level is related to the prognosis of patients.

Previous reports have established the best threshold of *MLL-*PTD at the initial diagnosis for predicting the CR or relapse in AML patients [13, 15]. However, the AML patients with *MLL-*PTD analyzed in above reports included both non-transplanted patients and transplanted patients. Since different treatments (chemotherapy and transplantation) have a great impact on the prognosis of AML patients, they also have a certain impact on the accuracy of the *MLL-*PTD threshold for predicting relapse. Allo-HSCT is one of the curative therapies currently available for AML and MDS-EB, so it is very necessary to establish an optimal threshold of post-transplant *MLL-*PTD for relapse in transplanted AML patients. In the analysis of the post-transplant best cut-off value, we found that *MLL-*PTD*/ABL* = 1% can be used as the threshold for predicting relapse. Based on this result, physicians could need to pay more attention to the occurrence of relapse for post-transplant patients with *MLL-*PTD*/ABL* ≥ 1%. Under this condition, it is also necessary to shorten the MRD monitoring interval, or give appropriate relapse preventive interventions in combination with the clinical condition.

A stable and reliable MRD marker whose expression level needs to vary with the tumor burden. Our data showed that *MLL-*PTD levels in relapsed patients were significantly increased before relapse. Importantly, there was no occurrence of *MLL-*PTD turning negative or losing before relapse, which indicated that *MLL-*PTD had a certain stability and could effectively reflect the change of tumor burden. As expected, *MLL-*PTD was available prior to hematological relapse, but the relapse after *MLL-*PTD positive occurred at different rates. One of the explanations may be due to the patient’s combination of additional mutations such as *FLT3-*ITD. Previous report confirms that *MLL-*PTD positive relapses harboring an additional *FLT3-*ITD mutation to relapse faster than other patients with *MLL*-PTD alone [15]. In our study, the initial diagnosis of 2 relapsed patients was accompanied by *FLT3-ITD* mutation. They respectively relapsed at 12 days and 35 days after post-transplant *MLL-*PTD*/ABL* ≥ 1%, and the relapse was significantly faster than that of other relapsed patients. These data suggested *MLL-*PTD patients with other mutations such as *FLT3-ITD* may need to be shortened intervals of MRD monitoring after transplantation. Of course, a larger sample size or data is needed in the future to further support the above result.

The timely monitoring of MRD in the early stage after transplantation was beneficial to guide early clinical intervention to improve the prognosis of patients. Some studies have confirmed that the *WT1* expression level is an independent prognostic indicator that can predict clinical outcome and combined use of *WT1* and flow cytometry monitoring can promote sensitivity of predicting relapse after allo-HSCT [21, 25]. For AML and MDS lacking specific markers, we usually need to combine FCM and *WT1* to evaluate MRD status. In the study, *MLL-*PTD became positive before relapse and prior to flow cytometry results. Thus, in contrast to FCM, PCR-based *MLL-*PTD detection have higher sensitivity. Our data showed that *MLL-*PTD seemed to be more sensitive than *WT1* in early MRD monitoring after transplantation. Furthermore, in contrast to *WT1*, *MLL-*PTD is more specific for the type of *MLL*-PTD positive AML and MDS. However, for post-transplant patients with *MLL-*PTD, in order to monitor MRD more effectively and accurately, there may not be a better way than monitoring FCM, *WT1* and *MLL-*PTD at the same time.

AML with *MLL-*PTD is a type of leukemia with a relatively poor prognosis compared with the standard-risk AML [13, 14]. In standard-risk AML, the post-transplant overall CIR and OS are around 15-20% and 60-70% at our institute, respectively [26, 27]. Our present results showed that the overall prognosis of post-transplant *MLL-*PTD patients (3-year OS: 67.8%; 3-year CIR: 13.7%) was similar to that of standard-risk patients. In addition, the other *MLL* rearrangement study about the transplant-related prognosis found that allo-HSCT would have a lower relapse risk and a higher survival probability compared to the results obtained from patients with chemotherapy alone [28]. The outcomes of patients with *MLL-*PTD are similar to the above results. The post-transplant OS in our study was significantly better than that of receiving chemotherapy alone (3-year OS< 40%) in previous study [5]. These data supported that allo-HSCT could achieve good therapeutic effect in patients with *MLL-*PTD at our institute. Furthermore, haplo-HSCT could achieve the similar therapeutic effect to the MSDT in patients with *MLL-*PTD. Therefore, our institution’s transplant and relapse prevention system may be effective for *MLL-*PTD patients.

## Conclusions

In conclusion, *MLL-*PTD expression is a sensitive and specific MRD marker for the *MLL-*PTD patients received allo-HSCT. *MLL-*PTD expression level higher than 1.0% suggested a high risk of hematological relapse and tended to have a worse prognosis. Furthermore, allo-HSCT could achieve good therapeutic effect in patients with *MLL-*PTD AML and MDS-EB. Of course, due to the limited number of patients with *MLL-*PTD patients, we still need to continue research to accumulate more cases to further confirm the significance of *MLL-*PTD for MRD monitoring around transplantation.

## Data Availability

The datasets used and analyzed during the current study are available from the corresponding author on reasonable request.

## Abbreviations

AML: Acute myeloid leukemia
MDS: Myelodysplastic syndrome
Allo-HSCT: Allogeneic hematopoietic stem cell transplantation
MRD: Minimal residual disease
RQ-PCR: Real-time quantitative polymerase chain reaction
TRM: Treatment-related mortality
CIR: Cumulative incidence of relapse
OS: Overall survival
DFS: Disease-free survival
*MLL*: Mixed-lineage leukemia
PTD: Partial tandem duplications
CR: Complete remission
MSDT: Matched sibling donor transplantation
NR: No remission
DLI: Donor lymphocyte infusion
BM: Bone marrow
FCM: Flow Cytometry
ANC: Absolute neutrophil count
aGVHD: Acute graft versus host disease
